# CPR-Induced Consciousness during Ventricular Fibrillation: Case Report and Literature Review

**DOI:** 10.1155/2024/2834376

**Published:** 2024-09-28

**Authors:** Xiaoqing Zhou, Boru Sun

**Affiliations:** Emergency Department Shengjing Hospital of China Medical University, Shenyang, Liaoning, China

## Abstract

**Introduction:**

Over the years, numerous studies have suggested the occurrence of a peculiar phenomenon known as “CPRIC” during the revival process. The revelation of this phenomenon has ignited widespread discussion and investigation, yet many enigmas remain unsolved. This study describes the case of a 52-year-old man diagnosed with acute anterior myocardial infarction, who experienced ventricular fibrillation while awaiting further treatment. Despite ultimately not regaining spontaneous circulation, he remained conscious for a period during chest compressions and showed signs of resistance.

**Methods:**

PubMed and Web of Science were searched until July 11, 2024. We included original studies and case reports relevant to CPRIC. For case reports, we extracted information on the author (year), country, patients, location, compression, signs of CPRIC, treatment of CPRIC, and patient outcomes. For other studies, we included the author (year), country, participants, and results. The extracted data were synthesized using a narrative approach.

**Results:**

Of 3038 articles, 32 were included, i.e., 18 case reports (24 cases), 9 cross-sectional surveys, and 5 cohort studies. In CPRIC cases, patients exhibited various manifestations including opening their eyes, speaking, and moving. Other included studies explored healthcare workers' awareness and experiences of CPRIC, the incidence and manifestations of CPRIC, the impact of CPRIC on patient outcomes, memories and perceptions of cardiac arrest indicating consciousness, the effects of CPRIC on rescuers, and the management of CPRIC.

**Conclusions:**

There is an urgent need to establish a globally recognized definition of CPRIC. It is crucial to develop clear algorithms that focus not only on identifying this phenomenon but also on determining the best approaches to manage it. Furthermore, CPRIC can cause multiple interruptions during CPR, making it essential to differentiate whether these interruptions are due to CPRIC or indicative of a return of spontaneous circulation.

## 1. Introduction

Cardiopulmonary resuscitation (CPR) is a critical emergency measure that provides life support for patients experiencing cardiac arrest. Recent research indicates that chest compressions can lead to a slight but crucial blood flow to the brain and sometimes, the cerebral perfusion pressure is sufficient to restore the patient's consciousness [1]. This phenomenon, known as CPR-induced consciousness (CPRIC), has triggered extensive discussions and studies, bearing significant importance for the medical community and emergency responders. In 1989, Lewinter et al. [2] first reported this phenomenon, and in 1994, Quinn et al. [3] further discussed CPRIC more explicitly. Although CPRIC-related cases have been reported, they remain relatively rare. An observational study of 16,558 out-of-hospital cardiac arrest patients found CPRIC in only 0.7% of the cases [4]. However, with improvements in prehospital systems and community response plans, the incidence of CPRIC is rising. One study found that the rate of CPRIC increased by 0.6% over 6 years [5]. In clinical practice, CPRIC appears to be more common than reported in the literature. A survey of 293 UK nurses revealed that 57% of the nurses had witnessed CPRIC, with over 56% experiencing it at least twice [6]. We presented a case report of a patient who developed signs consistent with CPRIC. We also describe the existing evidence regarding cognitive activity and consciousness during CPR, interventions involving sedation and analgesia during CPR, and the communication and ethical conflicts that CPRIC may engender.

### 1.1. Case Presentation

A male in his 50s, who was admitted to the hospital on June 9, 2023, with chest pain lasting for half an hour.

Diagnosis: acute anterior myocardial infarction.

ECG: ST elevation in leads V1–V4.

Clinically insignificant personal and familial history: denied hypertension, heart disease, and diabetes. No history of allergies, infectious diseases and epidemiology, or genetic diseases.

Biochemical and electrolyte data of this patient on emergency admission are summarized in Table 1.

21 : 43: emergency admission.

22 : 10: the first ventricular fibrillation, CPR.

Prompt manual chest compressions and defibrillation were initiated. Initially, the patient appeared extremely frightened and was able to open his eyes spontaneously but did not resist or speak. During the second round of chest compressions, despite attempts by the medical team to communicate, the patient resisted the intervention, claiming no noticeable change in his condition or discomfort. Although he could still open his eyes and speak, he remained in ventricular fibrillation when chest compressions were paused to check the rhythm. Family members and nurses intervened to prevent further resistance, but no restraints or sedation were administered.

22 : 12: return of spontaneous circulation (ROSC).

22 : 17: the second ventricular fibrillation, CPR, 25 defibrillations.

The patient remained conscious, could open his eyes spontaneously, and could speak words or phrases. He showed signs of distress and pain, struggling during the intervention. When compressions were paused to assess the rhythm, the patient remained in ventricular fibrillation. After compressions were stopped, he remained conscious briefly and then convulsed and became unconscious. Chest compressions resumed immediately. Due to the patient's awareness and resistance, rescuers replaced the electrode pads and reconnected them to rule out equipment malfunction. Throughout the resuscitation, the patient was neither restrained nor sedated; only the family and nurse held on his limbs. The medical team administered defibrillation, compressions, intermittent intravenous epinephrine, amiodarone, and dopamine for inotropic support. Despite experiencing asystole and confusion, chest compressions and defibrillation continued.

23 : 41: the doctor announced the patient's death, attributing it to myocardial infarction.

Throughout the resuscitation, the patient's family was present and expressed confusion, believing the patient was conscious. The medical team communicated with the family, explaining the reasons for the patient's agitated state and informing them of the severity of the situation. The family eventually understood and cooperated.

The rescuing doctor and nurses were encountering this situation for the first time and had only learned about cases of CPRIC through academic conferences. They had not received any specific training on how to handle such cases, which left them feeling perplexed.

## 2. Methods

### 2.1. Eligibility Criteria

We included original studies relevant to CPRIC (case reports, epidemiology, relationship with survival outcomes, impact on patients and rescuers, etc.) published in English, including but not limited to case reports, cross-sectional studies, cohort studies, and letters to editor. Journal articles, dissertations, conference abstracts, and conference proceedings were eligible. We excluded editorials and letters to the editor that presented author opinions or discussed controversies without reporting new phenomena or results.

### 2.2. Search Strategy

We searched PubMed and Web of Science using MeSH subject headings and free terms for articles published from inception to July 11, 2024. Detailed search strategies are presented in Table 2.

### 2.3. Study Selection and Data Extraction

After removing duplicates from the retrieved studies, we implemented a two-stage screening process to identify eligible articles. Initially, two independent reviewers evaluated the titles and abstracts based on predetermined eligibility criteria. Subsequently, the full texts of potentially relevant studies were reviewed independently. Any disagreements were resolved through discussion and consensus.

**Figure 1:**
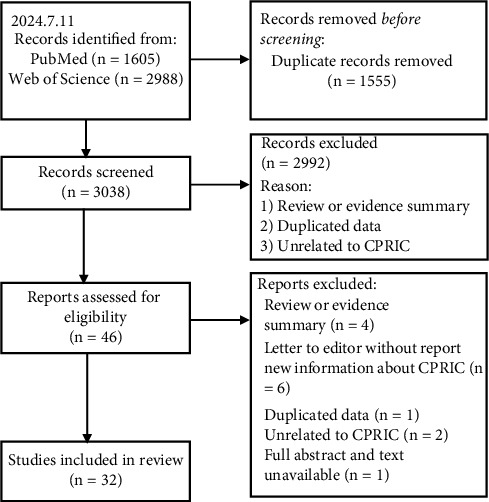
Flow diagram of the study selection process.

We retrieved a total of 4593 citations from electronic databases. After deduplication in EndNote X9, 3038 articles remained. Full text screening of 46 potentially eligible studies led to the inclusion of 32 studies (Figure 1) [2, 3, 5–34]. There included 24 journal articles [2, 3, 5–9, 11–15, 17–20, 22–27, 31, 32], 5 conference abstracts or proceedings [16, 28, 29, 33], and 3 letters to the editor [10, 21, 34]. Among the included studies, 18 reported 24 cases [2, 3, 7–22], 9 were cross-sectional surveys [6, 23–30], and 5 were cohort studies [5, 31–34]. These studies came from 16 different countries, with the United States contributing the largest number, totaling 7 articles. The publication dates of the included studies ranged from 1989 to 2024, with the largest number of publications in 2020 (3 articles).

We designed *s* data extraction form for case report, which included fields for author (year), country, patients, location, compression, signs of CPRIC, treatment of CPRIC, and patient outcome. For other studies, the form included fields for author (year), country, participants, and results. One reviewer used this form to extract data from all included studies, while another reviewer independently reviewed the extracted data.

## 3. Results

### 3.1. CPRIC Cases

The characteristics of included cases are shown in Table 3. Among the 24 cases, patient ages ranged from 22 to 89 years, 22 were male, and 2 were female. Twelve patients died, while 12 achieved ROSC. Most instances of CPR occurred in the emergency department (*n* = 13). In 3 cases, CPR was mechanical [2, 3, 11], and in 1 case, manual CPR was performed initially, followed by mechanical CPR [19]. When CPRIC occurs, patients exhibit a range of manifestations, including eye opening, speaking, and movement. In 13 cases, rescuers implemented measures to address CPRIC, such as physical restraint, communication with the patient, and intravenous administration of midazolam, succinylcholine, fentanyl, propofol, rocuronium, morphine sulfate, diazepam, and vecuronium.

### 3.2. Other Studies

In addition to case reports, the other included studies explored various aspects of CPRIC, including healthcare workers' awareness and experiences of CPRIC [6, 23, 24], the incidence and manifestations of CPRIC [5, 25, 29–34], the impact of CPRIC on patient outcomes [29–32], memories or perceptions of consciousness during cardiac arrest [26, 27], the effects of CPRIC on rescuers [28], and the management of CPRIC through cross-sectional surveys or cohort studies [5, 34], see Table 4. The following section will provide a detailed discussion of the included studies.

## 4. Discussion

### 4.1. The Definition and Characteristics of CPRIC

The exact features and scope of CPRIC remain unclear, and further research is needed to define them definitively. Some researchers define CPRIC as clinical symptoms of cerebral perfusion during CPR that do not occur when CPR is interrupted [35]. For example, Sukumar [17] reported cases where patients exhibited brief signs of consciousness such as eye opening, agitation, and resistance to rescuers' actions with their hands and head but did not respond to verbal commands. These signs disappeared when rescuers paused chest compressions to assess pulse and rhythm. Chin et al. [10] also provided a video demonstration of CPRIC.

While there is no universally accepted definition, CPRIC characteristics typically include spontaneous eye opening, movement of the arms, legs, or torso, speaking, obeying commands, experiencing pain, and even resisting CPR during cardiac arrest [14]. Most instances of CPRIC are temporary, occurring during the process of CPR, and reports of consciousness persisting throughout the entire procedure are rare [17]. However, not all cases of CPRIC exhibit overt signs of awareness. In an international multicenter observational study of 140 cardiac arrest survivors, 55 individuals (39%) reported feeling conscious during their period of coma but did not recall specific events related to resuscitation or other cognitive memories [27]. Among a subgroup of 101 survivors, 32 had cognitive memories, including feelings of fear. Nine survivors recalled memories consistent with near-death experiences, and two described explicit memories of visual and auditory events during CPR. Despite these recollections, there was no objective evidence of overt signs of consciousness, such as agitation, eye opening, or purposeful movements, in those patients, suggesting that consciousness may exist without overt signs of awareness. Some researchers proposed the following two forms of CPRIC [36]: interfering CPRIC (e.g., pushing or pulling rescuers away, moving limbs, pulling out intravenous tubing, biting on airway adjuncts, and talking to rescuers) and noninterfering CPRIC (e.g., blinking, breathing, eyes tracking, tearing, groaning, and slight movement). Different from the classification of Howard et al. [36], Parnia et al. [27] classified the above symptoms into one category, i.e., overt signs of consciousness, such as combativeness, groaning, and eye opening. The other group they classified encompasses symptoms that lack external signs of awareness but exhibits clear perceptual awareness and memory of visual and auditory stimuli. A systematic review encompassing reported cases found that consciousness in all instances included purposeful hand movements and could also involve painful breathing, eye opening, and responses to painful stimuli [37]. Furthermore, some patients engaged in detailed verbal and nonverbal communication with rescuers, and some were able to understand and comply with instructions. Others exhibited excitement and attempted to push rescuers away or expressed a desire to stop resuscitation. In reported cases, the Glasgow Coma Scale was used to assess the consciousness status of patients [17]. Some researchers divided the experience of CPR into the following four categories by establishing tests of visual and auditory awareness during CPR [26]: ① emergence from coma during CPR (CPRIC), ② in the post-resuscitation period, ③ dream-like experiences, and ④ transcendent recalled experience of death.

The incidence of CPRIC ranges from 0.23% to 0.9%, with combativeness or agitation being the most common symptom in 34.6% of the cases [5, 32]. It is estimated that 48–59% of “experienced” healthcare professionals report observing a patient with CPRIC during resuscitation [38]. However, it is currently unclear whether this high rate reflects the true prevalence of CPRIC or is a result of research design and small sample sizes. In a retrospective study involving 23,011 out-of-hospital cardiac arrest (OHCA) patients, 52 patients (0.23%) experienced CPRIC. This suggests that there were 2.3 cases of CPRIC per 1000 adult resuscitation attempts over a span of 12 years [32]. The most common sign of CPRIC was the presence of hostility/agitation, accounting for 34.6% of all cases (18 out of 52 cases).

CPRIC is more likely to occur in patients undergoing early CPR and defibrillation. Among patients experiencing CPRIC, a higher proportion of cases are witnessed by emergency responders, occur in public settings, have cardiac etiologies, and present with a shockable initial rhythm [32]. It is also more commonly observed in young male patients with ventricular fibrillation or pulseless ventricular tachycardia [12]. Reports of CPRIC occurring in older patients are relatively rare. For example, an 89-year-old male patient exhibited lower limb movements approximately 5 minutes after CPR was initiated [14]. These movements led to multiple interruptions in the resuscitation process; the first two interruptions were caused by the appearance of lower limb movements, prompting a pause in CPR to confirm the rhythm. Later, the patient attempted to push away the resuscitation provider, bit the endotracheal tube, showed signs of facial pain, and attempted to change his body position. After 24 minutes of CPR, the patient's signs of consciousness gradually decreased.

In summary, a clear definition of CPRIC is needed, which should extend beyond just agonal breathing. This definition should encompass the full range of behaviors and responses observed during CPR, including both overt and covert signs of consciousness. Future research should focus on identifying patients with a high probability of CPRIC and developing appropriate measures to address this phenomenon.

### 4.2. The Relationship between CPRIC with Survival

Some studies have indicated that patients with CPRIC have higher rates of spontaneous circulation recovery upon hospital admission, higher discharge survival rates, and better 30-day survival rates compared to those without CPRIC [32]. Although the study included a large sample size of 434 patients with CPRIC, it did not report whether these patients were treated with consciousness-altering drugs. The unadjusted odd ratio (OR) for ROSC with any sign of perfusion during CPR was 9 (95% CI: 3–24) [30]. Signs of life, such as gasping or respiratory movement, pupillary response, and movements during CPR, were associated with favorable outcome (OR: 11.0 and 95% CI: 3.7–32.5) [29]. However, CPRIC has not been confirmed as an independent predictor of survival. A study by Olaussen et al. [5] in 2017 found that in unwitnessed or bystander-witnessed events, CPRIC was independently associated with increased in-hospital survival (OR: 2.09 and 95% CI: 1.14–3.81), but this association was significant for patients who did not receive consciousness-altering drugs such as midazolam, opioids, and muscle relaxants. For patients who received consciousness-altering drugs, those who experienced CPRIC had a lower chance of survival compared to those who did not experience CPRIC (OR: 0.25 and 95% CI: 0.10–0.99). In this study, 37.5% patients with CPRIC received consciousness-altering drugs, whereas in the study by Doan et al. [32], only 6 patients (11.5%) did. Therefore, Doan et al. were more focused on patients who did not receive such treatments. From this perspective, the conclusions of these two studies are consistent. More research studies are needed to understand the impact of consciousness-altering drugs on patient survival and wellbeing. In conclusion, research in this area is still limited, and further empirical studies are required to reveal the underlying relationships.

### 4.3. The Mechanisms of CPRIC

Currently, research on the mechanisms underlying CPRIC remains quite limited, but several hypotheses have been proposed. One hypothesis suggests that the lack of oxygen and reduced cerebral blood flow during CPR may lead to changes in brain function, which may then be partially induced by CPR potentially giving rise to consciousness, and diminish once CPR is paused. Another hypothesis proposes that the cardiopulmonary stimulation during CPR may activate specific brain regions through neural pathways, leading to the generation of consciousness. Compressions can stimulate mechanoreceptors within the chest, which may activate neural reflex arcs involving the brainstem and other areas associated with arousal and consciousness. The reticular activating system (RAS) is essential for maintaining wakefulness, and mechanical stimulation from chest compressions may activate the RAS, leading to transient CPRIC. In addition, responses of the autonomic nervous system (ANS) during CPR may contribute to transient recovery of consciousness: During cardiac arrest and subsequent CPR, the body's stress response can trigger activation of the sympathetic nervous system, leading to increases in heart rate and blood pressure, which may enhance cerebral perfusion; vagus-mediated reflexes can also affect heart and brain function during CPR, potentially leading to transient recovery of consciousness.

Two studies have observed that an average arterial pressure exceeding 50 mmHg is sufficient to awaken patients [9, 18]. However, manual chest compressions rarely produce an average arterial pressure above 40 mmHg [39]. While it is possible to generate higher average arterial pressures, this does not necessarily lead to consciousness in all cases [40]. In addition, CPRIC can be influenced by individual factors such as autoregulation [41], ischemic threshold [42], and the presence of comorbidities [43], which may affect brain oxygenation levels.

Some researchers speculate that early and proficient CPR performed by trained personnel is a key factor contributing to CPRIC [9]. While mechanical devices have not demonstrated improved survival rates compared to manual chest compressions, there is evidence suggesting they play a role in enhancing the consistency of CPR, reducing interruptions [44] and improving cerebral perfusion pressure (CPP) compared to standard manual CPR [45]. Studies indicated that mechanical devices can generate 20–30% of prearrest cardiac output [46], which helps maintain cerebral perfusion pressure and potentially facilitates consciousness [18]. Manual CPR often involves brief interruptions per second, whereas mechanical CPR can provide continuous, uninterrupted compression. This consistent high-quality compression may be a key factor in the occurrence of consciousness. If uninterrupted CPR becomes more common, whether through increased use of mechanical CPR devices or improved manual CPR techniques, CPRIC may occur more frequently.

Another study reported CPRIC associated with resuscitative endovascular balloon occlusion of the aorta (REBOA) during CPR [20]. The researchers believe that REBOA, as an adjunct to CPR, may increase peripheral blood pressure [47] and aortic root blood pressure [48]. In the reported case, blood pressure increased during compressions, which may have led to enhanced cerebral perfusion and the occurrence of CPRIC. However, CPRIC is unlikely to be caused by increased blood pressure alone. Factors such as the patient's autoregulation, ischemic threshold, and comorbidities can influence cerebral oxygenation and thus the likelihood of achieving adequate cerebral perfusion for resuscitation consciousness.

### 4.4. The Psychological Effects of CPRIC on Both Patients and Rescuers

#### 4.4.1. The Psychological Effects on Patients

Purposeful movement or evidence of patient awareness during CPR may indicate high-quality cerebral perfusion resulting from effective resuscitation. However, conscious experiences occurring during CPR can lead to severe psychological consequences for survivors, which require careful attention and support [27]. An observational study found that 27% of the cardiac arrest survivors who experienced CPRIC developed post-traumatic stress disorder (PTSD) [49]. In a study of 101 cardiac arrest survivors, 46% of the patients recalled events following the cardiac arrest, with reported themes including fear, animals, plants, bright light, violence, déjà vu, and family [27].

Some researchers suggest conducting structured interviews and psychological counseling for all neurologically intact patients to understand the possibility of consciousness and symptoms of PTSD related to CPRIC [50, 51]. It is recommended to schedule interviews before patient discharge and continue them for six months [17]. Early cognitive-behavioral therapy in affected individuals can contribute to improving their psychological wellbeing. The latest American Heart Association guidelines do not cover sedation protocols or psychological assessments in the context of CPRIC [52]. There is almost no evidence to suggest that the use of sedatives or hypnotics can reduce or prevent the development of PTSD [17]. Large-scale sedation protocol trials are necessary to provide significant solutions regarding the psychological health and quality of life improvement for cardiac arrest survivors. It is important to note that while sedation may be used to alleviate psychological trauma, it must be balanced against the need to optimize patient survival and neurological outcomes. Sedation could potentially have negative effects on patient survival if not carefully managed, as it might interfere with hemodynamic stability and other critical physiological parameters. Therefore, if improved CPR quality indeed leads to favorable neurological recovery, a positive answer should be given to whether emphasizing sedation protocols to prevent post-traumatic psychological impacts in cases of CPRIC. Future research should focus on developing evidence-based sedation protocols that can mitigate psychological trauma without compromising survival outcomes.

Some researchers draw a parallel between CPRIC and Accidental Awareness during General Anesthesia (AAGA) [17]. AAGA refers to situations where a patient perceives their surroundings while considered to be in an unconscious state. AAGA is associated with connected consciousness. Currently, the understanding of the mechanisms of anesthesia or elimination of connected consciousness is primarily achieved through communication barriers between the prefrontal cortex and the thalamus, although it is suggested that pathways involving the thalamocortical, thalamo-reticular, intralaminar thalamic nuclei, mesencephalic, and brainstem regions also play important roles [53, 54]. Pharmacologically, this involves voluntary suppression of normally functioning and well-regulated central nervous system (CNS) before unconsciousness. In contrast, in patients experiencing cardiac arrest, the loss of consciousness is due to a lack of cerebral perfusion, and during CPR, the compromised perfusion is restored. This is a global cortical flow shutdown phenomenon, more related to hemodynamics. Although these mechanisms do not prove a direct analogy between AAGA and CPRIC, in both cases, subjective or objective measures of consciousness or implicit memories can lead to neurocognitive impairment. The consequences in both clinical scenarios may be comparable.

#### 4.4.2. The Psychological Effects on Rescuers

CPRIC can be a distressing experience for all parties involved. Patients may appear to be visually distressed, leaving traumatic memories for clinical staff and bystanders. Two case reports described how rescue personnel felt disturbed and anxious for a considerable period after experiencing a CPRIC event [8, 55]. In an observational study, over 90% of the participants reported adverse effects of CPRIC, with 52% experiencing personal discomfort and 7% reporting insomnia, nightmares, and mood changes [28]. Surveys also indicated that rescuers experienced distress and tension when encountering CPRIC, particularly when healthcare providers were unfamiliar with this phenomenon [6]. Another interviewee discussed the discomfort felt by a healthcare provider who participated in a CPRIC case, where the patient deliberately moved their arms and occasionally sat up, attempting to push away the hands performing chest compressions. In addition, rescuers may also feel confused or distracted. Some individuals lacked awareness and training regarding CPRIC, while others were puzzled by how to manage a patient's physical responses during cardiac arrest [24].

### 4.5. The Management of CPRIC

Rescuers' reports regarding disruptions to CPR attempts due to CPRIC include instances where patients resisted chest compressions or attempted to remove vascular access devices, necessitating a pause in CPR to comfort the patient. In some cases, sedatives or paralytic medications were administered, and physical restraints were used to prevent further interference [6, 25, 28]. In addition, patients were instructed not to touch the endotracheal tube to ensure the effectiveness of the airway management during resuscitation [18].

There are varying practices concerning the use of sedatives and analgesics during CPRIC. Brede et al. [23] suggested fentanyl, ketamine, and midazolam as the most appropriate sedation agents. In studies of Olaussen et al. [25] and Grandi et al. [11], sedatives and analgesics were administered during resuscitation. On the other hand, studies by Rice et al. [56], Ulrichs et al. [57], and Pound et al. [15] used only sedative drugs. In the case reported by Pinot et al. [14], sedatives were not used because they were unsure of their impact on the success of CPR; they solely relied on physical restraints. However, the authors suggest that ketamine is the most beneficial drug in such cases. Some researchers recommend the use of fentanyl or ketamine for patients who achieve a certain level of consciousness while mechanical compression devices are in operation [19]. A study in 2017 found that among 112 CPRIC patients, 42 received treatment with sedatives, muscle relaxants, opioids, or a combination of these drugs [5]. The sedative strategies used commonly were midazolam (0.1 mg·kg^−1^) + succinylcholine (1.5 mg·kg^−1^) [3], and “small doses” of morphine sulfate + diazepam [2]. However, midazolam has negative inotropic effects [58], which raises legitimate concerns about potential harm, changes in perfusion characteristics, and a negative correlation with discharge rates associated with its use.

It can be anticipated that agents causing minimal circulatory depression would be the preferred choice, as this particular concern is a significant factor that may deter clinicians from administering sedation during resuscitation [9]. Despite the relatively limited impact of advanced life support medications on cardiac arrest outcomes [59, 60], there is still a concern that sedative doses could potentially affect patient survival. A study found that the use of sedation or analgesia was associated with worse outcomes, including an increase in termination of on-scene resuscitation efforts, prolonged time to achieve ROSC, and a decrease in in-hospital survival rates [37].

While there is no universal International Liaison Committee on Resuscitation (ILCOR) guideline, the Dutch guideline for prehospital cardiac arrest suggests using 2 *μ*g·kg^−1^ of intravenous fentanyl (which can be titrated to 4 *μ*g·kg^−1^) for pain relief during mechanical chest compressions, along with 2.5 mg of intravenous midazolam (which can be titrated to 5 mg) for sedation [61]. Other regions also allow the use of small doses of sedatives to facilitate endotracheal intubation in cases with a vomiting reflex [62]. Howard et al. [63] summarized the guidelines for prehospital management of CPRIC. However, these guidelines are not extensively supported by evidence, but they have the potential to provide fewer interruptions during CPR. Lundsgaards [64] also conducted an evidence review on pain management in patients experiencing CPRIC and found limited scientific evidence supporting protocols for pain management during CPR. Further research is needed to support the selection of medications in such situations.

Regarding how to distinguish consciousness resulting from ROSC from CPRIC, the utilization of intra-arrest point-of-care ultrasonography has the potential to provide immediate and dynamic information regarding the response to medical interventions and assess intrinsic cardiac activity. When combined with pulse checks, point-of-care ultrasonography can significantly enhance the accuracy in identifying perfusing rhythms [65].

Rescuers who have experienced CPRIC indicated that CPRIC poses a treatment challenge to the resuscitation team and that an effective CPRIC management strategy is essential to guide the resuscitation team in making informed decisions [22]. Currently, Sedation methods should be both effective and humane, while considering potential adverse effects. In the prehospital emergency environment, physiological monitoring may be relatively basic, thus guidelines in such settings may require a more conservative approach. In addition, these CPRIC patients are being managed with highly diverse techniques, and there is no research on how these differences might impact the rate of ROSC.

### 4.6. Communication and Ethical Conflicts Arising from CPRIC

When CPRIC is evident, resuscitation efforts are influenced by bystanders. Bystanders may question whether the patient truly experienced cardiac arrest and may believe that the patient is improving. If the patient does not ultimately survive, this can easily lead to medical-ethical conflicts. When CPRIC disrupts resuscitation, including pushing and grabbing at rescuers, terminating compressions, and pulling on endotracheal tubes and mechanical devices, communication between patients and rescuers becomes more challenging. In a systematic review, in half of the reported cases, patients were able to push rescuers away, leading to interruptions in the CPR process [37]. Even when patients survive, there may be a sense of aversion towards the rescuers. For example, surviving patients may think that rescuers were trying to hold them down and leaved them with nowhere to escape [66]. Therefore, ethical considerations are essential when dealing with patients exhibiting signs of distress.

CPRIC is extremely important for emergency physicians because various ethical issues may arise [67]. Currently, there is an ethical issue concerning whether individuals exhibiting CPRIC have the capacity to make decisions about their treatment. In a medical context, capacity is defined as the ability of an individual to utilize information about their medical condition and proposed treatment options to make choices that align with their preferences [68]. However, in patients undergoing CPR, it is challenging to determine whether they truly comprehend and reason to make decisions. Lundsgaards [69] designed a qualitative, descriptive study to explore the perspectives of cardiac arrest team members regarding communication and ethical conflicts related to CPRIC. However, the authors did not report on the outcomes of the interviews.

## 5. Future Directions

CPRIC is a phenomenon that has sparked extensive discussions and research. CPRIC involves various forms of consciousness during CPR, with symptoms ranging from overt signs such as eye opening and movement to covert awareness without external indicators, but its exact characteristics and scope remain unclear. The incidence of CPRIC was generally less than 1% in various surveys. CPRIC patients who did not receive consciousness-altering medications have been shown to have higher survival rates than patients who do not develop CPRIC. There is currently a lack of evidence on whether the use of mechanical CPR equipment increases the incidence of CPRIC. Among patients with CPRIC, nearly one third of surviving patients would develop PTSD. In addition to being an interruption for rescuers, CPRIC can cause them to feel uneasy and anxious for a long time after the incident.

There are currently no universally accepted guidelines regarding the types and doses of sedative and analgesic medications that can be used during resuscitation of patients with CPRIC. Most researchers agreed on the use of ketamine [1, 36]. Due to concerns about the impact of ketamine on perfusion in patients with high shock index [70], further studies are needed to demonstrate whether the use of this drug will change the prognosis of patients with CPRIC. The authors advocate finding a dose that can quickly and effectively sedate patients suffering from CPRIC and prevent the patient's behavior from affecting defibrillation operations and distracting rescuers. There is a lack of clear consensus on the second-line drugs and doses for the treatment of interfering CPRIC, especially the use of fentanyl and midazolam.

In addition, research should actively answer whether sedation protocols can prevent post-traumatic psychological effects in CPRIC cases. Beyond this, whether individuals with CPRIC have the capacity to make decisions about their treatment is an ethical issue that requires active discussion. It is also necessary to think about how to make CPRIC patients realize that the rescuer's operation is to save their lives so as not to resist rescue operations. Last but not least, how to best identify patients at risk for CPRIC and how to implement strategies to deal with them if they are at greater risk are also a critical direction.

## 6. Conclusion

CPRIC, though rare with an incidence of less than 1%, poses significant clinical and ethical challenges. It affects not only the patients, who may develop PTSD, but also the rescuers, who may experience prolonged anxiety. There is a lack of consensus on the use of sedatives and analgesics during resuscitation, with ketamine being a commonly recommended option, though its impact on patients with a high shock index remains uncertain. Further research is needed to establish effective sedation protocols, prevent post-traumatic psychological effects, and address the ethical implications of patient awareness during CPR. Identifying patients at risk for CPRIC and developing appropriate management strategies are critical future directions.

## Figures and Tables

**Table 1:** Biochemical and electrolyte data summary.

Items	Value	Normal range
*Blood gas analysis*		
pH	7.41	7.35∼7.45
PaCO_2_ (mmHg)	38	35∼45
PaO_2_ (mmHg)	70 ↓	75∼100
HCO_3_- (mmol/L)	24.1	22∼26
Temp	37.0	—
K+ (mmol/L)	2.9 ↓	3.5∼5.5
Glu (mmol/L)	13.2	
Na+ (mmol/L)	137	135∼145
Lac (mmol/L)	1.8	
Ca++ (mmol/L)	1.09 ↓	1.15∼1.29
Hct (%)	43	35∼50
HCO_3_std (mmol/L)	24.6	22∼26
TCO_2_ (mmol/L)	25.3	24.0∼32.0
BEecf (mmol/L)	−0.5	−3∼3
Ca++(7.4) (/LP)	1.09 ↓	1.15∼1.29
THbc (g/L)	146	120∼180
SO2c (%)	94 ↓	95∼98
BE(B) (mmol/L)	−0.3	−3∼3

*Troponin I*		
Tropl (*μ*g/L)	0.0212 ↑	0∼0.0198

*PRO-BNP*		
PRO-BNP (pg/mL)	20.54	<125

*Blood routine examination*		
WBC (10^9^/L)	16.6 ↑	3.5∼9.5
NEUT (%)	47.5	43.2∼71.5
LYMPH (%)	44.5 ↑	16.8∼43.4
MONO (%)	6.3	4.6∼12.4
EO (%)	1.1	0.7∼7.8
BASO (%)	0.1 ↓	0.22∼1.21
NEUT (10^9^/L)	7.9 ↑	1.9∼7.2
LYMPH (10^9^/L)	7.4 ↑	1.1∼2.7
MONO (10^9^/L)	1.0 ↑	0.3∼0.8
EO (10^9^/L)	0.2	0.04∼0.49
BASO (10^9^/L)	0.0 ↓	0.01∼0.1
RBC (10^12^/L)	5.33	4.3∼5.8
HGB (g/L)	144	130∼172
HCT (%)	41.5	40∼52
MCV (fL)	77.9 ↓	83∼101
MCH (pg)	27.0 ↓	27.2∼34.7
MCHC (g/L)	347	329∼360
RDW-CV (%)	12.4	11.6∼14.4
RDW-SD (fL)	34.5 ↓	35.1∼46.3
PLT (10^9^/L)	410 ↑	135∼350
PDW (fL)	10.2	10.00∼15.00

**Table 2:** Search strategies and results for each database from inception to Jul. 11, 2024.

Database	Results	Search strategies		Results
PubMed	2024.7.11	#1	(“Awareness”[Mesh]) OR “(Perception”[Mesh]) OR “(Consciousness”[Mesh]) OR “(Pain Perception”[Mesh]) OR “(Wakefulness”[Mesh])	1605
		#2	(“Consciousness”[Title/Abstract] OR “Awareness”[Title/Abstract] OR “Perception”[Title/Abstract] OR “Pain perception”[Title/Abstract] OR “Movement”[Title/Abstract] OR “Wakefulness”[Title/Abstract] OR “combative”[Title/Abstract] OR “alert”[Title/Abstract] OR “awake”[Title/Abstract])	
		#3	#1 OR #2	
		#4	(“Cardiopulmonary Resuscitation”[Mesh])	
		#5	(“CPR”[Title/Abstract] OR “cardiopulmonary resuscitation”[Title/Abstract] OR “cardiopulmonary resuscitations”[Title/Abstract] OR “resuscitation cardiopulmonary”[Title/Abstract] OR “heart resuscitation”[Title/Abstract] OR “cardio pulmonary resuscitation”[Title/Abstract] OR “cardiac resuscitation”[Title/Abstract])	
		#6	#4 OR #5	
		#7	(“CPR-induced consciousness”[Title/Abstract])	
		#8	(#3 AND #6) OR #7	

Web of Science	2024.7.11	((TS = (“Consciousness” OR “Awareness” OR “Perception” OR “Pain perception” OR “Movement” OR “Wakefulness” OR “combative” OR “alert” OR “awake”)) AND TS = (“CPR” OR “cardiopulmonary resuscitation” OR “cardiopulmonary resuscitations” OR “resuscitation cardiopulmonary” OR “heart resuscitation” OR “cardio pulmonary resuscitation” OR “cardiac resuscitation”)) OR TS = (“CPR-induced consciousness”) and article or other or case report or abstract or meeting or dissertation thesis or clinical trial or early access or reference material or book or bibliography or biography (document types)		2988

**Table 3:** Characteristics of included CPRIC cases described in detail.

Author (year)	Country	Patients		Location	Compression device	Signs of CPRIC	Treatment of CPRIC	Patient outcome
		Age (years)	Sex					
Al Atbi et al., (2022)	Oman	49	Male	Emergency room	—	(1) Verbalizing (2) Moving: pulling the hands away from his chest, kicking foot	(1) Midazolam 0.1 mg/kg (2) Succinylcholine 1.5 mg/kg	Dead

Asghar et al., (2020)	Pakistan	62	Male	ICU	—	(1) Eyes remained open (2) Obeyed simple commands (e.g., to open his mouth)	—	Dead

Bihari and Rajajee, (2008)	India	57	Male	General medical ward	Manual	(1) Tracked the surrounding health care providers with eyes (2) Moving both his arms very purposefully (brisk localization); attempted to localize and resist chest compressions, to pull the laryngoscope away as chest compressions were in progress (3) One and a half hours after initiating chest compressions he remained conscious, opening eyes to call and moving both hands appropriately to command	(1) Manually immobilized	Dead

Chin et al., (2020)	China	42	Male	Ambulance	Manual	(1) Made noises and purposeful movements	—	ROSC

Grandi et al., (2017)	Italy	87	Male	Emergency room	Manual	(1) Purposeful movements, attempt to grab forearms of CPR performer	(1) Physical restraint (2) Fentanyl	ROSC
		80	Male	Emergency room	Manual	(1) Purposeful movements, attempt to remove hands of CPR performer	(1) Physical restraint	ROSC
		82	Male	Emergency room	Manual	(1) Open eyes (2) Scream (3) Attempt to remove hands of CPR performer	(1) Physical restraint (2) Fentanyl	Dead
		50	Male	Emergency room	Mechanical	(1) Respiratory movements during compressions	—	Dead
		22	Male	Emergency room	Manual	(1) Ocular movements (2) Incomprehensible sounds	(1) Propofol (2) Rocuronium	ROSC
		84	Male	Emergency room	Manual	(1) Shout during administration of shock	(1) Propofol	ROSC

Gray (2018)	Canada	38	Male	Emergency room	Manual	(1) Made purposeful movements to push CPR providers away (2) Verbalized with defibrillations	(1) 4-point restraint	ROSC

Lewinter et al., (1989)	US	60	Female	Emergency room	Mechanical	(1) Responsive	(1) Small doses of IV morphine sulfate (2) Small doses of diazepam	Dead

Morais et al., (2024)	Brazil	59	Male	Out-of-hospital	Manual	(1) Moving his upper limbs in an attempt to remove the bag-valve-mask device	—	ROSC

Pinto et al., (2020)	Portugal	89	Male	Emergency room	—	(1) Presented lower limb movements, tried to push away the CPR provider with his hands, bit endotracheal tube, showed facial signs of pain, and tried to move sideways	—	Dead

Pound et al., (2017)	Canada	52	Male	Out-of-hospital	Manual	(1) Eyes open (2) Moaning and yelling (3) Moving all limbs in what appeared to be purposeful movement to stop the resuscitation efforts (push away the CPR compressor); actively biting on the laryngoscope and swinging his arms toward his face during ongoing CPR	(1) Midazolam 2 mg	ROSC

Quinn et al., (1994)	Canada	57	Male	Emergency room	Mechanical	(1) Became agitated, violently moving his arms and legs; purposeful movement of hands (i.e., pulling at the ACD device and ventilation mask)	(1) Limb restraints (2) Midazolam 0.1 mg/kg (3) Succinylcholine 1.5 mg/kg	Dead

Singh et al., (2020)	USA	64	Female	Hospital	—	(1) Demonstrated purposeful movements (2) Verbalized a desire to halt CPR	—	Dead

Sukumar (2019)	India	52	Male	Emergency room	Manual	(1) Had transient signs of consciousness such as opening eyes, agitation, and resisting the rescuer with his hands and head movements (2) No response to verbal commands	—	ROSC

Tobin and Mihm, (2009)	USA	62	Male	Hospital	Manual	(1) Slightly open eyes (2) Made inspiratory efforts, moved his head; reached for the endotracheal tube (3) After told that he was receiving life-sustaining chest compressions, the patient appeared to understand this and refrained from reaching for the endotracheal tube again. He was now able to wiggle his toes and give a “thumbs up” to command	(1) Communicated with patients	Dead

Wacht et al., (2015)	Israel	57	Male	Out-of-hospital	Manual—mechanical	(1) Moving his hands toward the compression device and grabbed it; had folded his hands and would not allow the paramedics to straighten them	—	ROSC

Brede and Skjærseth, (2023)	Norway	71	Male	Ambulance	Manual	(1) Normalized breathing efforts at 12–15 breaths per minute (2) Eye opening without ROSC	—	Dead

Hoppenfeld et al., (2016)	USA	50	Male	PACU	—	(1) Remained conscious, demonstrating purposeful movements (2) Breathing spontaneously	—	ROSC
		51	Male	PACU	—	(1) Attempting to push the staff away	—	ROSC

Ilyas et al., (2023)	India	55	Male	Emergency room	—	(1) After 40 minutes of resuscitation, when it seemed like all probability of resuscitation would fail, the patient started moving with the flexing and extending of both hands and legs with the opening of the eyes	(1) Restraining him with their hands (2) Midazolam 3 mg (3) Vecuronium 4 mg	Dead

**Table 4:** Characteristics of other included studies.

Author (year)	Country	Participants	Results
Brede et al., (2024)	Norway	115 physicians working at air ambulance, search-and-rescue base or physician-staffed rapid response car	(1) 88 heard of CPRIC prior to survey (2) 105 experienced CPRIC

Gregory et al., (2021)	UK	293 paramedics who were registered with the Health and Care Professions Council and working in the United Kingdom	(1) 167 had witnessed CPRIC; of those, over 56% had experienced it on at least two occasions (2) CPRIC was deemed to interfere with resuscitation in nearly 50% of first experiences but this fell to around 31% by the third experience (3) The most common reasons for CPRIC to interfere with resuscitation were patient resisting clinical interventions, increased rhythm and pulse checks, distress, confusion, and reluctance to perform CPR

Mays et al., (2019)	UK	293 paramedics who were registered with the Health and Care Professions Council and working in the United Kingdom	(1) Over 50% had heard of CPRIC (2) Over 40% became aware of CPRIC after having witnessed it in clinical practice

Olaussen et al., (2016)	Australia	100 experienced health care professionals, including doctors, nurses, and paramedics	(1) Most responders (59 of 67) to the question had experienced CPR-noninterfering consciousness and reported experiencing it a median of 3 (IQR: 1–5) times (2) CPR-interfering consciousness had been experienced by 51 of the 63 responders and was experienced overall 1 (IQR: 1–3) time (3) Management of these cases varied widely with ranging from no action to sedation and/or paralysis (4) A guideline describing the management of this presentation was considered necessary by 40 out of 57 (70%) responders

Parnia et al., (2023)	USA	567 in-hospital cardiac arrest patients	(1) 11 reported cardiac arrest memories/perceptions suggestive of consciousness; (2) Four categories of experiences emerged: ① emergence from coma during CPR (CPR-induced consciousness), ② in the postresuscitation period, ③ dream-like experiences, and ④ transcendent recalled experience of death
		126 community cardiac arrest survivors	(1) Reinforced the categories identified by study 1 and identified another: delusions

Parnia et al., (2014)	USA, UK, Austria	(1) 140 cardiac arrest survivors-stage 1 interviews (2) 101 of 140 cardiac arrest survivors in stage -stage 2 interviews	(1) 46% had memories with 7 major cognitive themes: fear, animals/plants, bright light, violence/persecution, deja-vu, family, recalling events post-CA (2) 9% had NDEs, while 2% described awareness with explicit recall of “seeing” and “hearing” actual events related to their resuscitation

Versteeg et al., (2019)	Netherlands	71 ANE, ED, ICU physicians	(1) 48% reported multiple experiences with CPRIC and >90% reported (detrimental) effects on treatment and team-members. While 52% reported personal discomfort, 7% reported sleeplessness, nightmares, and mood changes, extending up to weeks (2) all exposed reported frustration in remediation

Debaty et al., (2018)	France	437 patients treated with extracorporeal CPR	(1) Signs of life were observed in 261 (59%) patients, with 136 (31%) patients presenting gasping or respiratory movement, 155 (35%) a pupillary response, and 49 (11%) movements during CPR (2) Factors associated with favorable outcome were: signs of life during CPR (OR: 11.0), first recorded rhythm VF/VT (OR: 3.4), low-flow duration per min (OR: 0.99)

Moore et al., (2018)	USA	102 patients treated with extracorporeal CPR	(1) Improved color during CPR was seen in 23/102 (23%), pulse during CPR in 17/102 (17%), gasping in 18/102 (18%), and movement during CPR in 5/102 (5%) (2) The unadjusted OR for any sign of perfusion during CPR for a CPC score of 1 or 2 was 26 and for any sign of perfusion during CPR for ROSC was 9

Debaty et al., (2021)	France	434 extracorporeal CPR recipients in 2010s	(1) The prevalence of any sign of life was 61%: pupillary light reaction (48%), gasping (32%), and increased level of consciousness (13%) (2) The adjusted odds ratios of 30-day survival with favorable neurological outcome were 7.35, 5.86, 4.79, and 1.75 for any sign of life, pupillary light reaction, increased level of consciousness, and gasping, respectively

Doan et al., (2020)	Australia	23011 out-of-hospital cardiac arrest patients from 2007 to 2018	(1) 52 (0.23%) were CPRIC (2) Combativeness/agitation was the most common sign of CPRIC (34.6%) (3) CPRIC patients had numerically higher rates of return of spontaneous circulation on hospital arrival, discharge survival, and 30-day survival, than those without CPRIC; however, CPRIC was not found to be an independent predictor of survival

Olaussen et al., (2017)	Australia	Adult out-of-hospital cardiac arrest patients treated by emergency medical services (EMS) from 2008 to 2014	(1) There were 112 (0.7%) cases of CPRIC among 16,558 EMS attempted resuscitations, increasing in frequency from 0.3% in 2008 to 0.9% in 2014 (2) Levels of consciousness consisted of spontaneous eye opening (20.5%), jaw tone (20.5%), speech (29.5%), and/or body movement (87.5%) (3) CPRIC was independently associated with an increased odds of survival to hospital discharge in unwitnessed/bystander witnessed events but not in EMS witnessed events (4) Forty-two (37.5%) patients with CPRIC received treatment with one or more of midazolam (35.7%), opiates (5.4%), or muscle relaxants (3.6%) (5) CPRIC in unwitnessed/bystander witnessed patients was associated with improved odds of survival to hospital discharge if medications were not given but did not influence survival if these medications were given

Parnia et al., (2013)	USA, UK, Austria	2060 cardiac arrest events, 152 survivors were interviewed	(1) Information on visual or auditory impressions during CA was available on 89% of interviewed subjects; of these, 37% responded that they had experienced visual and/or auditory impressions during their time of unconsciousness and CA (2) among those with auditory and/or visual impressions, 70% had a greyson score >0 and 30% had a greyson score ≥7, consistent with a conventionally defined “NDE”

Talikowska et al., (2024)	Australia	6803 out-of-hospital cardiac arrest with EMS -attempted resuscitation from 2018.1 to 2023.12	(1) Identified 42 CPRIC cases (0.62%) (2) CPRIC was classified as “interfering” with OHCA management (3) The most common CPRIC manifestations were “purposeful movement” (64%, *n* = 27) and “taking breaths” (55%, *n* = 23); (4) Intravenous ketamine was administered for CPRIC in 3 (7%) cases

## Data Availability

Data sharing is not applicable to this article as no new data were created or analyzed in this study.

## Abbreviations

CPR: Cardiopulmonary resuscitation
ROSC: Return of spontaneous circulation
CPRIC: Cardiopulmonary resuscitation-induced consciousness
OHCA: Out-of-hospital cardiac arrest
CPP: Cerebral perfusion pressure
PTSD: Post-traumatic stress disorder
AAGA: Accidental awareness during general anesthesia
CNS: Central nervous system
ILCOR: International liaison committee on resuscitation
REBOA: Resuscitative endovascular balloon occlusion of the aorta.

## References

[1] Dąbrowski S., Lange S., Basiński A. (2023). Analgesic use in patients during cardio-pulmonary resuscitation. *International Journal of Environmental Research and Public Health*.

[2] Lewinter J. R., Carden D. L., Nowak R. M., Enriquez E., Martin G. B. (1989). CPR-dependent consciousness: evidence for cardiac compression causing forward flow. *Annals of Emergency Medicine*.

[3] Quinn J. V., Hebert P. C., Stiell I. G. (1994). Need for sedation in a patient undergoing active compression--decompression cardiopulmonary resuscitation. *Academic Emergency Medicine*.

[4] Pourmand A., Hill B., Yamane D., Kuhl E. (2019). Approach to cardiopulmonary resuscitation induced consciousness, an emergency medicine perspective. *The American Journal of Emergency Medicine*.

[5] Olaussen A., Nehme Z., Shepherd M. (2017). Consciousness induced during cardiopulmonary resuscitation: an observational study. *Resuscitation*.

[6] Gregory P., Mays B., Kilner T., Sudron C. (2021). An exploration of UK paramedics’ experiences of cardiopulmonary resuscitation-induced consciousness. *British Paramedic Journal*.

[7] Al Atbi A. Y. H., Al Mandhari A., Al Reesi A. (2022). Cardiopulmonary resuscitation induced consciousness: a case report. *Oman Medical Journal*.

[8] Asghar A., Salim B., Tahir S., Islam F., Khan M. F. (2020). Awareness during cardiopulmonary resuscitation. *Indian Journal of Critical Care Medicine*.

[9] Bihari S., Rajajee V. (2008). Prolonged retention of awareness during cardiopulmonary resuscitation for asystolic cardiac arrest. *Neurocritical Care*.

[10] Chin K. C., Yang S. C., Chiang W. C. (2020). Video of cardiopulmonary resuscitation induced consciousness during ventricular fibrillation. *Resuscitation*.

[11] Grandi T., Carlo S., Carosi V. (2017). Six cases of CPR-induced consciousness in witnessed cardiac arrest. *Italian Journal of Emergency Medicine*.

[12] Gray R. (2018). Consciousness with cardiopulmonary resuscitation. *Canadian Family Physician*.

[13] Morais D. A., Moura A. D., Guelfi D. C. F., Moraes C. M. Gd, Machado G. A. C. (2024). Cardiopulmonary resuscitation induced consciousness: case report. *International Journal of Cardiovascular Sciences*.

[14] Pinto J., Almeida P., Ribeiro F., Simões R. (2020). Cardiopulmonary resuscitation induced consciousness A case report in an elderly patient. *European Journal of Case Reports in Internal Medicine*.

[15] Pound J., Verbeek P. R., Cheskes S. (2017). CPR induced consciousness during out-of-hospital cardiac arrest: a case report on an emerging phenomenon. *Prehospital Emergency Care*.

[16] Singh R. P., Adhikari S., Landsberg D., Kaul V. (2020). Cardiopulmonary resuscitation-induced consciousness. *Baylor University Medical Center Proceedings*.

[17] Sukumar V. (2019). Having a conscious patient during cardiopulmonary resuscitation: is it not time to consider sedation protocol?: a case report. *A&A Practice*.

[18] Tobin J. M., Mihm F. G. (2009). A hemodynamic profile for consciousness during cardiopulmonary resuscitation. *Anesthesia and Analgesia*.

[19] Wacht O., Huri R., Strugo R. (2015). Case Study: combative Cardiac Patient. What do you do when a patient regains consciousness during mechanical CPR?. *EMS World*.

[20] Brede J. R., Skjærseth E. (2024). Resuscitative endovascular balloon occlusion of the aorta (REBOA) during cardiac resuscitation increased cerebral perfusion to occurrence of cardiopulmonary resuscitation-induced consciousness, a case report. *Resuscitation*.

[21] Hoppenfeld M. S., Kotov A., Ortega R. (2016). Ventricular fibrillation and consciousness are not mutually exclusive. *Resuscitation*.

[22] Ilyas W. M., Gadkari C., Singh A., Chavan G. (2023). A rare phenomenon of pulseless body movements induced during prolonged cardiopulmonary resuscitation. *Cureus*.

[23] Brede J. R., Skjærseth E., Rehn M. (2024). Prehospital anaesthesiologists experience with cardiopulmonary resuscitation-induced consciousness in Norway - a national cross-sectional survey. *Resuscitation*.

[24] Mays B., Gregory P., Sudron C., Kilner T. (2019). Awareness of CPR-induced consciousness by UK paramedics. *British Paramedic Journal*.

[25] Olaussen A., Shepherd M., Nehme Z. (2016). CPR-induced consciousness: a cross-sectional study of healthcare practitioners’ experience. *Australasian Emergency Nursing Journal*.

[26] Parnia S., Keshavarz Shirazi T., Patel J. (2023). AWAreness during REsuscitation - II: a multi-center study of consciousness and awareness in cardiac arrest. *Resuscitation*.

[27] Parnia S., Spearpoint K., de Vos G. (2014). AWARE-AWAreness during REsuscitation-a prospective study. *Resuscitation*.

[28] Versteeg J., Noordergraaf J., Vis L., Willems P., Bremer R. (2019). CPR-induced consciousness: attention required for caregivers and medication. *Resuscitation*.

[29] Debaty G., Nicol M., Aubert R. (2018). Abstract 362: early signs of life as a prognostic factor for extracorporeal cardiopulmonary resuscitation in refractory out-of-hospital cardiac arrest. *Circulation*.

[30] Moore J. C., Grahl M., Marko T. (2018). Abstract 113: signs of perfusion during cardiopulmonary resuscitation as noted by first responders is predictive of good neurologic outcome in cardiac arrest. *Circulation*.

[31] Debaty G., Lamhaut L., Aubert R. (2021). Prognostic value of signs of life throughout cardiopulmonary resuscitation for refractory out-of-hospital cardiac arrest. *Resuscitation*.

[32] Doan T. N., Adams L., Schultz B. V. (2020). Insights into the epidemiology of cardiopulmonary resuscitation-induced consciousness in out-of-hospital cardiac arrest. *Emergency Medicine Australasia*.

[33] Parnia S., Fenwick P., Spearpoint K. (2013). Abstract 236: a multi center study of awareness during resuscitation. *Circulation*.

[34] Talikowska M., Belcher J., Ball S., Majewski D., Finn J. (2024). CPR-induced consciousness in out-of-hospital cardiac arrest patients in Western Australia: case characteristics and CPR quality. *Resuscitation*.

[35] Greb C., Heightman A. J. (2014). Mechanical CPR helps save the day–and the patient. https://www.jems.com/patient-care/mechanical-cpr-helps-save-day-and-patien/.

[36] Howard J., Grusd E., Rice D. (2024). Development of an international prehospital CPR-induced consciousness guideline: a Delphi study. *Paramedicine*.

[37] Olaussen A., Shepherd M., Nehme Z., Smith K., Bernard S., Mitra B. (2015). Return of consciousness during ongoing cardiopulmonary resuscitation: a systematic review. *Resuscitation*.

[38] West R. L., Otto Q., Drennan I. R. (2022). CPR-related cognitive activity, consciousness, awareness and recall, and its management: a scoping review. *Resuscitation*.

[39] Nolan J. P., Soar J., Zideman D. A. (2010). European resuscitation council guidelines for resuscitation 2010 section 1. Executive summary. *Resuscitation*.

[40] McDonald J. L. (1982). Systolic and mean arterial pressures during manual and mechanical CPR in humans. *Annals of Emergency Medicine*.

[41] Moppett I. K., Hardman J. G. (2007). Modeling the causes of variation in brain tissue oxygenation. *Anesthesia and Analgesia*.

[42] Bandera E., Botteri M., Minelli C., Sutton A., Abrams K. R., Latronico N. (2006). Cerebral blood flow threshold of ischemic penumbra and infarct core in acute ischemic stroke: a systematic review. *Stroke*.

[43] Jespersen S. N., Østergaard L. (2012). The roles of cerebral blood flow, capillary transit time heterogeneity, and oxygen tension in brain oxygenation and metabolism. *Journal of Cerebral Blood Flow and Metabolism*.

[44] Ong M. E., Mackey K. E., Zhang Z. C. (2012). Mechanical CPR devices compared to manual CPR during out-of-hospital cardiac arrest and ambulance transport: a systematic review. *Scandinavian Journal of Trauma, Resuscitation and Emergency Medicine*.

[45] Metzger A. K., Herman M., McKnite S., Tang W., Yannopoulos D. (2012). Improved cerebral perfusion pressures and 24-hr neurological survival in a porcine model of cardiac arrest with active compression-decompression cardiopulmonary resuscitation and augmentation of negative intrathoracic pressure. *Critical Care Medicine*.

[46] Martens P., Mullie A. (1995). Sedation during and after CPR-efforts: is it worth a guideline?. *Resuscitation*.

[47] Brede J. R., Skjærseth E., Klepstad P., Nordseth T., Krüger A. J. (2021). Changes in peripheral arterial blood pressure after resuscitative endovascular balloon occlusion of the aorta (REBOA) in non-traumatic cardiac arrest patients. *BMC Emergency Medicine*.

[48] Jang D.-H., Lee D. K., Jo Y. H., Park S. M., Oh Y. T., Im C. W. (2022). Resuscitative endovascular occlusion of the aorta (REBOA) as a mechanical method for increasing the coronary perfusion pressure in non-traumatic out-of-hospital cardiac arrest patients. *Resuscitation*.

[49] Gamper G., Willeit M., Sterz F. (2004). Life after death: posttraumatic stress disorder in survivors of cardiac arrest--prevalence, associated factors, and the influence of sedation and analgesia. *Critical Care Medicine*.

[50] Merikle P. M., Daneman M. (1996). Memory for unconsciously perceived events: evidence from anesthetized patients. *Consciousness and Cognition*.

[51] Cruse D., Chennu S., Chatelle C. (2011). Bedside detection of awareness in the vegetative state: a cohort study. *The Lancet*.

[52] Panchal A. R., Bartos J. A., Cabañas J. G. (2020). Part 3: adult basic and advanced life support: 2020 American heart association guidelines for cardiopulmonary resuscitation and emergency cardiovascular care. *Circulation*.

[53] Hudetz A. G., Mashour G. A. (2016). Disconnecting consciousness: is there a common anesthetic end point?. *Anesthesia and Analgesia*.

[54] Demertzi A., Soddu A., Laureys S. (2013). Consciousness supporting networks. *Current Opinion in Neurobiology*.

[55] McDonald G. (2005). Code blue stories. Awake and aware in the emergency department. *Hospital Physician*.

[56] Rice D. T., Nudell N. G., Habrat D. A., Smith J. E., Ernest E. V. (2016). CPR induced consciousness: it’s time for sedation protocols for this growing population. *Resuscitation*.

[57] Ulrichs C. J., Böttiger B. W., Padosch S. A. (2014). Total recall--is it ethical not to sedate people during successful resuscitation?. *Resuscitation*.

[58] Matsuura N. (2017). Muscle power during intravenous sedation. *Japanese Dental Science Review*.

[59] Lin S., Callaway C. W., Shah P. S. (2014). Adrenaline for out-of-hospital cardiac arrest resuscitation: a systematic review and meta-analysis of randomized controlled trials. *Resuscitation*.

[60] Nolan J. P., Perkins G. D. (2013). Is there a role for adrenaline during cardiopulmonary resuscitation?. *Current Opinion in Critical Care*.

[61] Ambulancezorg M. (2023). Landelijk protocol ambulancezorg. https://www.scholamedica.nl/images/VB01-2023-LPA9-is-nu.pdf.

[62] Ambulance V. (2014). *Ambulance Victoria Clinical Practice Guidelines for Ambulance and MICA Paramedics/Ambulance Victoria*.

[63] Howard J., Lipscombe C., Beovich B. (2022). Pre-hospital guidelines for CPR-Induced Consciousness (CPRIC): a scoping review. *Resuscitation*.

[64] Lundsgaard R. S., Lundsgaard K. S. (2019). Bet 2: pain management in patients who show awareness during CPR. *Emergency Medicine Journal*.

[65] Zengin S., Yavuz E., Al B. (2016). Benefits of cardiac sonography performed by a non-expert sonographer in patients with non-traumatic cardiopulmonary arrest. *Resuscitation*.

[66] Fauber J. (2011). New CPR devices save lives, Medical College study finds. https://archive.jsonline.com/features/health/114171424.html/.

[67] Varon J. (2019). Awareness during resuscitation: where is the data?. *The American Journal of Emergency Medicine*.

[68] Barstow C., Shahan B., Roberts M. (2018). Evaluating medical decision-making capacity in practice. *American Family Physician*.

[69] Lundsgaard R. S., Lundsgaard K. S. (2018). Cardiac arrest teams perspectives on communication and ethical conflicts related to awareness during CPR, a focus group study protocol. *Scandinavian Journal of Trauma, Resuscitation and Emergency Medicine*.

[70] Miller M., Kruit N., Heldreich C. (2016). Hemodynamic response after rapid sequence induction with ketamine in out-of-hospital patients at risk of shock as defined by the shock index. *Annals of Emergency Medicine*.

